# Inside Out: Change Through Art

**DOI:** 10.1089/whr.2020.0092

**Published:** 2020-10-22

**Authors:** Marcia B. Haffmans, Edward O. McFalls

**Affiliations:** ^1^ Department of Medicine, University of Minnesota, Minneapolis, Minnesota, USA.; ^2^ Division of Cardiology, VA Medical Center, Minneapolis, Minnesota, USA.

**Keywords:** art activism, family health, incarceration, stress

## Abstract

***Background:*** Over the past three decades, there has been a 900% increase in the number of women experiencing incarceration in Minnesota. We wished to test whether handwriting, as creative visual art expression for women in jail, would be a positive experience for them as well as for individuals viewing the artwork during expositions.

***Methods:*** Over a 2-year period, the principal artist invited women residents from four separate county jails in Minnesota, to handwrite their thoughts on a sheet of paper. Two hundred twenty-three women residents participated in the artist-led handwriting/visual art sessions and gave permission to use their authentic script, anonymously, for presentation in a 3-D visual art form. At the conclusion of the sessions, a survey was offered at each venue, which asked three questions relative to the participation in the handwriting art project: (1) Did it have a positive impact on me? (2) Would you recommend it to other women who are incarcerated? (3) Do you want to participate in more projects such as this during your incarceration? The resulting artwork of more than 1,000 sculptures, each exhibiting a portion of the women's original script, was displayed at several public showings and a survey was offered at each venue, which asked: (1) Did the exhibition increase awareness of mass incarceration of women? (2) Did it help the viewer see women who are incarcerated? (3) Did it make the viewer realize that action is needed to reduce incarceration of women? Survey questions were graded from 1 to 5, with a sliding scale from strongly disagree to strongly agree.

***Results:*** The impact of this project of art activism has been very positive on both participants and the larger audience. The vast majority of women residents responding to the survey either agreed or strongly agreed that their participation in the project (1) had a positive effect on them (94%), (2) would recommend it to other incarcerated women (94%), and (3) would want to participate in more projects such as this (93%). A total of 425 surveys were collected among the audience at several sites: the law school (*N* = 87), open studios (*N* = 268), and a public library (*N* = 62). The vast majority of individuals responding to the survey either agreed or strongly agreed that the exhibited work (1) increased awareness of the problem (93%), (2) showed the humanity behind the script (88%), and (3) suggested that interventions were needed to address the problem (86%).

***Conclusions:*** Women under incarceration in county jails, who participated in a visual art handwriting program, as part of a collaborative visual art project led by principal artist, found great value in the sessions and agreed that such programs should be available to other women in detention. The overwhelming majority of the audience of the resulting exhibitions in public venues strongly agreed that interventions are needed to address the mass incarceration of women in Minnesota, suggesting the importance of art as a vehicle for increasing awareness about social problems and perhaps social change.

## Introduction

Art exhibits have been shown to be an important format to highlight shared values among patients and providers in the health care system^1^ and, when used in public venues, can serve as a valuable tool to address inequities of diverse members of our community.^2,3^ Empathy, expressed through the arts, may be one of the most important ways that our society can reduce polarization between individuals with different backgrounds. This is particularly true in the present times, where the novel COVID-19 virus coupled with excessive police force against BIPOC (Black, Indigenous, People of Color) has marginalized many unfortunate members of our communities. The combination of COVID-19 among individuals incarcerated in jail led to grave implications about the welfare of citizens in urban America.^4^

Women create strong foundational forces in communities and we need to ensure that all women have a chance to leverage their unique sensibilities to create harmony and sustainability in their neighborhoods and places of employment, so that we all can survive and flourish. This project was stimulated by the rate of incarceration among women in Minnesota, which has increased nearly ninefold over the past 30 years. The effects on their lives as primary caregivers to their children need to be recognized.^5^ Although the reasons for this escalation of mass incarceration of women are multifactorial, it is clear that the inequities within the judicial system related to mass incarceration, particularly among BIPOC and poor women, need better scrutiny.^6^ We need action on all fronts to eradicate the control of the criminal justice system affecting more than 1,000,000 women nationwide.

Art evokes empathy, and this format may be one of the most important tools to address the need for social change.^7,8^ Using art forms of authentic handwritings from women under incarceration, the goal of this project was twofold. First, we wished to determine whether the implementation of scheduled art projects with women in jail could bring value to their lives. Second, we sought to determine whether demonstrating their art forms, highlighting their individual handwritings, could bring value to the viewing public. To determine whether the art project provided benefits, we offered questionnaires to both the women creating the art and the public viewing the authentic handwritings in art installations.

## Methods

### Art sessions with women experiencing incarceration

Between July 2017 and December 2019, the principal artist was granted the right to work with women experiencing incarceration in four separate county jails in Minnesota, lasting 1–2 hours/session. Of note, requests to offer art sessions by the principal artist to the Minnesota State as well as Federal Women's prisons had been formally denied. The goal was to engage the women by handwriting their thoughts and explore art techniques that expanded on the concept of handwriting as visual art, utilizing printmaking, painting, sculpting with clay, and embroidery techniques. At the conclusion of each workshop, a voluntary, anonymous survey was offered to every participant at each of the venues, and >90% of them responded to the questionnaires. The survey asked three questions about the session, including (1) Did it have a positive impact on me? (2) Would you recommend it to other women who are incarcerated? (3) Do you want to participate in more projects such as this during your incarceration? Each survey question was given a numerical response from 1 to 5, enumerating Strongly Disagree to Strongly Agree and a space for comments was available. At the conclusion of all sessions, the forms were collected and the data were entered into an Excel File for further analysis.

### Art exhibits of handwritings of women experiencing incarceration

The principal artist applied the handwritings and incorporated them in art installations at six public venues serving different communities. At each of the public showings, a survey was offered to viewers, which asked three questions relative to the art exposition, including (1) Did it increase my awareness of mass incarceration of women? (2) Did it help me see women who are incarcerated? (3) Did it make me realize that action is needed to reduce incarceration of women? Each survey question was given a numerical response from 1 to 5, enumerating Strongly Disagree to Strongly Agree and a space for comments was available. At the conclusion of all sessions, the forms were collected and the data were entered into an Excel File for further analysis.

## Results

### Art workshops with women experiencing incarceration

**FIG. 1.**
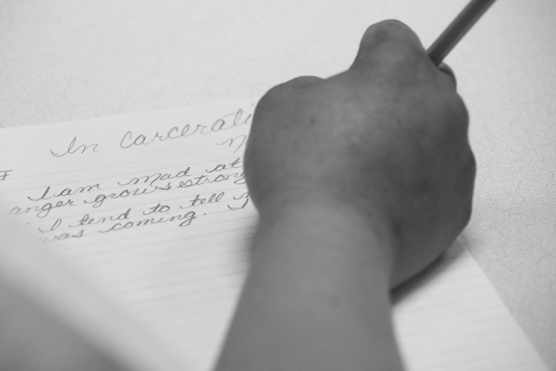
Handwriting during workshop. A woman participant in one of the jails is creating expressive handwriting as part of the visual art class.

**FIG. 2.**
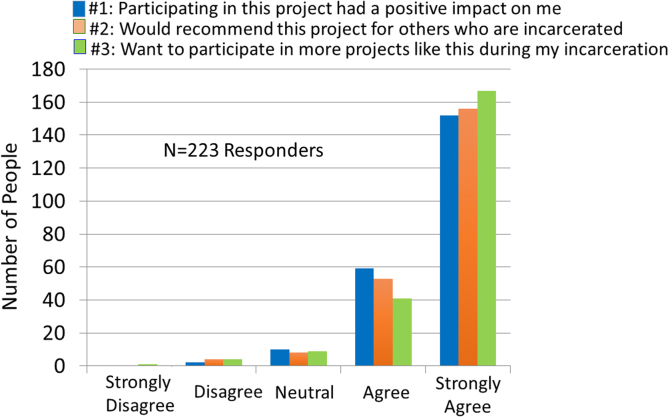
Summary data from the questionnaires obtained from the 223 women at the four jails are shown for each of the questions. The overwhelming response was viewed as favorable for each of the questions.

Among the 4 county jails, 223 women residents participated in the voluntary artist-led handwriting/visual art classes and used their own handwritings to express their ideas (Fig. 1). At the conclusion of the sessions, they were offered a questionnaire and on a voluntary basis, provided feedback by completing the surveys. Based on the number of women in the workshops and the return of the surveys, completion was available for >90% of the women who participated in the classes. The vast majority of individuals responding to the survey either agreed or strongly agreed that their participation in the project (1) had a positive impact on them (94%), (2) would recommend it to other women who are incarcerated (94%), and (3) would want to participate in more projects such as this (93%). The data from each of the jails are summarized in Table 1 and Figure 2. A list of all comments from the women is provided in Table 2.

**Table 1.** Summary of Survey Responses from the Women in Minnesota Jails

	Strongly disagree	Disagree	Neutral	Agree	Strongly agree
Question #1					
Wright County (*N* = 151)	0	0	7	39	105
Ramsey County (*N* = 32)	0	2	3	12	15
Hennepin County (*N* = 23)	0	0	0	3	20
Goodhue County (*N* = 17)	0	0	0	5	12
Question #2					
Wright County (*N* = 151)	0	0	5	36	110
Ramsey County (*N* = 32)	0	4	2	13	11
Hennepin County (*N* = 23)	0	0	0	3	20
Goodhue County (*N* = 17)	0	0	1	1	15
Question #3					
Wright County (*N* = 151)	0	1	4	29	117
Ramsey County (*N* = 32)	1	3	5	9	13
Hennepin County (*N* = 23)	0	0	0	2	21
Goodhue County (*N* = 17)	0	0	0	1	16
Total (*N* = 223)	1	4 (1%)	9 (4%)	41 (18%)	167 (75%)

**Table 2.** Verbatim (unedited) Transcribed Handwritten Comments from Participants of Art Workshops in Four Minnesota Jails

From the Incarcerated Women's Survey @ Ramsey Co Correctional Facility, St. Paul (& Goodhue Co Adult Detention Center, Red Wing^*^) (*N* = 32)
[^*^7 surveys from Goodhue mixed in with Ramsey]
Question #1: “*Participating in this project had a positive impact on me*”
• “I wasn't expecting the words to be so deep”
• “this has motivated me to do more art”
• “we all have a voice”
• “We all need help”
• “This should be practiced more”
• “It made me more open with myself”
• “Enjoyable”
• “My voice on paper”
• “I would love to Contive this more!”
• “I believe women should help motivate one another no matter where they come from”
Question #2: “*I would recommend this project for others who are incarcerated*”
• “Should be @ Shakopee prison”
• “the pictures meant a lot to me”
• “It gave me a sense of carefreeness”
• “you can inspire anybody”
• “Very uplifting”
• “You can make a Possitive Change (4) Yowself”
• “Yes I would”
Question #3: “*I want to participate in more projects like this one during my incarceration*”
• “And outside of prison”
• “Thank you”
• “pretty please”
• “this was surprising and exciting to me”
• “Painting”
• “Love this!”
• “It would be an honor to see more!”
• “Freedom”
• “Very inspirational”
• “Help mental state of mind”
• “recovery matters to me”
• “Indeed, after also”
• “They should offer these projects everywhere!”
From the Incarcerated Women's Survey @ Goodhue Co Adult Detention Center, Red Wing (*N* = 17)
Question #1: “*Participating in this project had a positive impact on me*”
• “mind opening”
• “gives me something to look forward to”
• “made me rethink the negative of why I'm here”
• “keeps me believing others care!”
• “able to express myself.”
Question #2: “*I would recommend this project for others who are incarcerated*”
• “could help others open their minds too”
• “yes!”
• “it helps express your feelings in different ways”
• “interesting!”
• “It's supportive & optimistic”
• “it's therapeutic”
Question #3: “*I want to participate in more projects like this one during my incarceration*”
• “I'll come while I can”
• “yes, yes, yes!”
• “it's eye opening & beautiful”
• “I would be honored to do more”
• “it's therapeutic”
From the Incarcerated Women's Survey @ Wright Co. Jail, Buffalo (*N* = 151)
Question #1: “*Participating in this project had a positive impact on me*”
• “I wasn't expecting the words to be so deep”
• “therapeutic”
• “Feeling—yes—TY”
• “made me feel again”
• “It's helping me know that it's ok to express myself”
• “This is cool”
• “Everyone can share something”
• “Makes me feel ”
• “I have a voice!”
• “I've been doing more writing since this class”
• “Thank you”
• “Art is so important when you have no one”
• “PLEASE GIVE US FREEDOM THROUGH ART”
• “Very inspiring”
• “very interesting”
• “Learn that you can express yourself through art”
• “It helped my mind wander with creativity”
• “Love it”
• “Very nice lady”
• “Amazing group”
• “I really enjoyed everything about this project”
• “Grateful”
• “I feel people forget about people in jail, + you are showing the world!”
• “It was a fantastic experience”
• “You're amazing”
• “Thank you for sharing your look on the article world, eyes”
• “Yes, I look forward to coming to class each week”
• “Yes, I like learning new & different ways of art”
• “really enjoyed it”
• “it's pretty cool”
• “lots of positivity + open ideas”
• “definitely”
• “LOVE IT!”
• “you make it unique”
• “I could express myself in a creative way”
• “was very wonderful”
• “Loved it!”
• “learned a lot”
• “very relaxing but brings the creativity out in me”
• “It was very cathartic for me.”
• “I LOVE this class”
• “More of a hands on learner”
• “I love this class”
• “It's a positive outlet”
• “Thank you”
• “Very moving”
• “Appreciate programming during incarceration”
• “[Smiley Face]”
• “And other women smile”
• “I love this class!”
• “Thank you, it helps me to feel a part”
• “Release my expression's awareness”
• “Really like”
• “She was an amazing teacher”
Question #2: “*I would recommend this project for others who are incarcerated*”
• “as a release form for healing”
• “Yes – still a+ inspirational”
• “very helpful + interesting”
• “this is awesome”
• “Give it one time”
• “Ur treasured”
• “It helps people open up + write about whatever they feel”
• “Very cool”
• “This helped me forget where I was and made me feel important”
• “Yes, different”
• “Give you a stress reliever”
• “Immensely”
• “Love it”
• “Interesting project”
• “Influence positive”
• “Feel like someone cares”
• “I talked about it all week long”
• “Put a smile on my face”
• “Thank you for sharing your article's outlook on life”
• “I great outlet!”
• “Thank you for sharing”
• “Definitely, need more programs like this”
• “yes”
• “yes!”
• “inspiring”
• “very different from other programs”
• “yes I would”
• “Highly!”
• “definitely”
• “Very positive”
• “This project is so fun and informative”
• “I think almost all the women would love this project”
• “I brought other women today”
• “[Smiley Face]”
• “Show people on the outside what we experience”
• “Yes I feel our voice is heard”
• “It allowed me to feel my hurts”
• “It gets your voice heard”
• “I invite everyone I can”
• “It's good interaction. Positive”
• “Gives understanding of our inside outlet”
• “I love this class!”
• “Freedom of mind, stress relief, helps me express”
• “Yes”
Question #3: “*I want to participate in more projects like this one during my incarceration*
• “Help me understand I too can be an artist”
• “Finally!! Something worth doing in jail”
• “Thank you”
• “gives different insight”
• “Thank you”
• “very nice lady. I enjoyed attending”
• “Enjoying & appreciate the art past/present”
• “helps with mind”
• “It's very educational because you find out different places to visit that have amazing art with handwriting + other interesting pieces in it”
• “I would come back daily, weekly + monthly”
• “GETS ME OUT”
• “Blessed be”
• “I think this is very helpful for our writing skills”
• “Positive outlook and healing”
• “I would love to see you again”
• “There is a healing element that comes with art”
• “Anything to keep me busy, thank you”
• “Its fun”
• “Absolutely”
• “Love it”
• “Love to”
• “Painting”
• “Thank you for your help”
• “Always looking for NEW”
• “Loved it, thank you”
• “Anything”
• “Interesting, fun!”
• “I think this is a great writing class I will come again”
• “This is my 2nd time coming”
• “Yes!”
• “Thank you again! I have never looked at life like that!”
• “I am grateful for the opportunity to escape, even if it's only for a few moments!”
• “Amazing”
• “Yes makes you think”
• “Yes, I'll come back to this class”
• “Yes would help lots of people”
• “yes beneficial”
• “Knowledge is great skill”
• “Every day!”
• “Very beneficial”
• “Thank you Marcia!”
• “You inspired me”
• “It allows me to forget and let myself be creative for a little bit.”
• “I am going to share how great and fun this class is. Please come back as much as possible.”
• “I wish you could come more often”
• “I have ADHD—hard 2 stay focused—will continue 2 attend!”
• “I love art”
• “It helps me to focus on a future for me. Thank you”
• “Yes I was finally heard thru art and felt very moving”
• “Open-minded to be a volunteer in the future”
• “Thank You [Smiley Face]”
• “I strongly agree because sometimes when you feel like you have no one around you and even by putting pen to paper it allows me to ”
• “Expression, freedom of our minds, dreams, thoughts”
• “It's educational, mental health self grounding therapeutic, ease during a dark place”
• “I love this class!”
• “Amazing vibes”
• “Yes very interesting”
• “I'm leaving”
From the Incarcerated Women's Survey @ Hennepin Co. Adult Correctional Facility, Plymouth (*N* = 23)
Question #1: “*Participating in this project had a positive impact on me*”
• “it made me feel strong”
• “Motivating + uplifting”
• “I feel like what I am saying matters”
• “So much talent”
• “open my eyes more”
• “”
• “Love it”
• “yes good vibige”
• “interesting”
• “love it”
• “I love it!”
• “yes very inspiring”
• “Awesome class”
• “Good & Cultured”
• “I totally love the energy”
Question #2: “*I would recommend this project for others who are incarcerated*”
• “You are listened to, words are being taken into consideration”
• “Marcia's passion is contagious! Remarkable”
• “it's a very good thing”
• “To be respectful”
• “yes”
• “”
• “Yes made me ”
• “yes, I would”
• “for sure | A must”
• “yes!!!”
• “yes most definitely”
• “Love it”
• “Relaxing”
• “Her intentions are pure!”
Question #3: “*I want to participate in more projects like this one during my incarceration*”
• “Looking forward to her return”
• “yes”
• “Makes my time easier, able to get things off my chest.”
• “So that I could learn more”
• “yes I would love that”
• “”
• “very helpful”
• “yes, please”
• “Yes I would like to”
• “Please—much love”
• “It's a great outlet”
• “I certainly would”
• “I LOVE THIS CLASS”
• “I appreciate art ”

The principal artist kept a diary for short notes written immediately after each session. It shows that an art form introduced by the principal artist of crumpled paper, bound tight together and suspended with a long string, hovering above the gallery floor, led to a participant's exclamation, “that is exactly how I feel.” Through their own handwritten visual art, participants expressed feelings of frustration, angst, anger, isolation, and more. Mothers frequently wrote about their children, how they deserved better than their parent in jail. There are chronological stories of “why” and poems about “here.” People imagined a safe home, free from abuse, reciprocated love, and work opportunities with childcare options, instead of drug dependency and mental health challenges. Most wrote about the importance of family that they have been barred from. Less than a handful out of all stories addressed how they finally had reached safety, including food and shelter; however, the majority stated that life was on hold in an unpleasant place with uncertainty for survival on release. Unedited submissions contained erasures and spelling bamboozles, with expressions such as “I am a rose without pedals,” “I lost my self-esteam to myself,” or “I am greatful to finally be leaving.” Others described physical and mental agony, in addition to loss of freedom: “hundreds of others have worn the clothing,” “changed my meds to suite the jail to make money,” and “I am wasting sitting here doing Nothing! Making no money!.” Many expressed faith and “everything happens for a reason.” Altogether, the writings offer deep insight and possible solutions that support lives rather than control them, including access to free counseling for mental health and substance addiction, living wage jobs, affordable childcare, housing, nutrition, and education. The project empowered the women by validating their feelings and self-actualizing their voice. The cries for self-realization need the attention of all community members for policy changes from within the affected group to explore ways that achieve self-fulfillment without state control.

### Art exhibits of handwritings of women experiencing incarceration

**FIG. 3.**
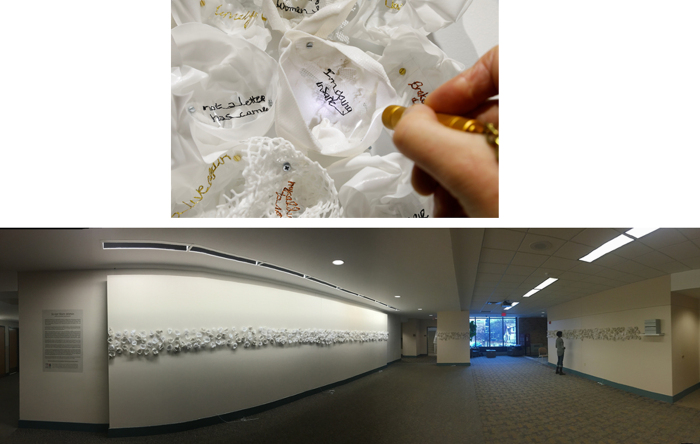
Installation art of handwritings. An example of an art installation is shown in detail and overview at a local law school. The original artwork of the handwritings has been embedded into the sculptures, for site-specific installations.

**FIG. 4.**
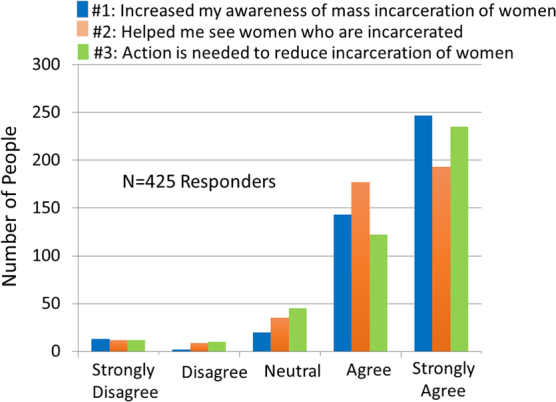
Summary data from the questionnaires obtained from the 425 respondents at the 6 public exhibition venues are shown for each of the questions. The overwhelming response was viewed as favorable for each of the questions.

Portions of the authentic handwritten reflections were traced by the principal artist through hand-stitching onto transparent polymer and embedded into artist-made individual 3-D sculptures for viewing. More than 1,000 sculptures, each exhibiting a portion of the women's original script, were created and exhibited in different configurations at each of the venues. An example of one of the art installations, at a local law school, is shown in Figure 3. A total of 425 surveys were collected, including those from exhibitions at law school (*N* = 87), open studios (*N* = 268), and a public library (*N* = 62). An informal counting of individuals viewing the art shows during open studios estimated that 10%–20% of viewers provided feedback by completing the surveys. The vast majority of individuals responding to the survey either agreed or strongly agreed that the exhibited work (1) increased awareness of the problem (93%), (2) showed the humanity behind the script (88%), and (3) suggested that action is needed to address the problem (86%). The data from all public showings are summarized in Table 3 and Figure 4, and a complete list of additional comments in the survey is provided in Table 4.

**Table 3.** Summary of Surveys from the Public Viewers at Six Art Venues

	Strongly disagree	Disagree	Neutral	Agree	Strongly agree
Question #1					
Art-a-Whirl (*N* = 140)	1	0	3	60	76
Open Casket (*N* = 64)	2	0	0	25	37
Mitchell Law (*N* = 87)	4	0	4	19	60
Amersfoort NL (*N* = 15)	0	0	1	6	8
Walker Library (*N* = 62)	2	1	5	12	42
Phipps Exhibit (*N* = 57)	4	1	7	21	24
Question #2					
Art-a-Whirl (*N* = 140)	0	2	19	73	46
Open Casket (*N* = 64)	2	0	3	31	28
Mitchell Law (*N* = 87)	3	2	1	23	59
Amersfoort NL (*N* = 15)	0	0	3	5	7
Walker Library (*N* = 62)	3	1	4	25	29
Phipps Exhibit (*N* = 57)	4	4	5	20	24
Question #3					
Art-a-Whirl (*N* = 140)	1	2	14	49	73
Open Casket (*N* = 64)	2	0	4	19	40
Mitchell Law (*N* = 87)	3	3	10	16	55
Amersfoort NL (*N* = 15)	0	0	2	4	9
Walker Library (*N* = 62)	2	2	6	17	34
Phipps Exhibit (*N* = 57)	4	3	9	17	24
Total (*N* = 425)	12 (3%)	10 (2%)	45 (11%)	122 (29%)	235 (55%)

**Table 4.** Verbatim (unedited) Transcribed Handwritten Comments from Viewing Audience at Six Public Exhibitions

From the Public's Survey at Law School (*N* = 87)
Question #1: “*This exhibit has increased my awareness of mass incarceration of women*”
• “already aware”
• “Had no idea. Looks kind of messy”
• “I thought the exhibit would be more. I feel women have a lot more to share”
• “already quite aware”
• “900%! What! WOW!”
• “How many are victims?!”
• “I currently work with incarcerated women”
• “was not aware of severity, % of mothers”
• “Love the facts in the beginning”
• “since I work at Women's Prison Book Project, my awareness is already high and I am already violently anti-prison”
• “Yes, I learned about the facts locally, nationally and worldwide.”
• “POWERFUL”
• “I had never realized how many women were incarcerated in MN before this”
• “WOW!”
• “Mind boggling statistics—for women incarcerated in US”
• “I was quite aware”
• “More about MN (I knew a lot going in)”
• “your facts were critical to learn about”
• “Eye opening”
• “so many lost souls”
• “very well done”
• “it's amazing how much emotion can come from 2–4 words”
• “the struggle to read the handwriting reminded me of the struggle to write it”
• “I have already been aware—this is emotional reinforcement. EXCELLENT”
• “very interesting”
• “what a powerful testament. The loneliness, hurt, pain—so real!”
• “moving, emotional”
• “Thank you for this”
• “Very moving”
• “Thank you!”
• “900% increase is unconscionable”
• “Your life means something”
• “wish I knew more!!”
• “didn't realize 900% increase”
• “thank you so much for educating us about their ster..”
• “much more is needed”
• “tragic”
• “It really opened my eyes to the conditions of the prison system in America and how it is disproportional to the African American and minority communities.”
Question #2: “*This exhibit has helped me see women who are incarcerated*”
• “would have liked to read some whole letters”
• “no idea”
• “beautifully done”
• “They miss what we hardly notice—sun, moon, grass”
• “This made me very sad”
• “Absolutely!”
• “Yes, I really saw their voices in their writing and got a feeling for their experience”
• “definitely”
• “we need to humanize and not demoralize people in prison”
• “very sensitive”
• “the handwriting was eye-opening”
• “they are part of the world—visible”
• “it is very sad”
• “I lingered more on certain pieces, but they were represented equally—no bias”
• “is there remorse”
• “Thank you! Very simple but powerful.”
• “couldn't get away from the reality”
• “Very profound”
• “Thank you”
• “It's a drug problem”
• “Your story matters”
• “very insightful”
• “powerful words”
• “some of the comments were so sad”
• “I was not aware of the conditions people go through”
• “would love to see a more larger form of narrative”
• “I don't know anyone who has been incarcerated”
• “delicate holding of profound prayers, angst, and love”
Question #3: “*This exhibit has made me realize that action is needed to reduce incarceration of wom*en”
• “already know, but thank you”
• “There should be an approach against incarceration in general”
• “I knew that already but really appreciate the exhibit”
• “Somehow we need to teach HOPE”
• “Their children are punished and families broken up for smoking marijuana!!”
• “Felt the spirits of the women”
• “Powerful show. The repetitive nature of forms is also powerful and makes me sad. Loved it!”
• “Incredible exhibit! Thanks you for sharing with us!”
• “900% is terribly frightening”
• “Your work helps people see how justice is a dialogue. Beautiful!”
• “Yes-how?”
• “Thank you for your work”
• “varies on conduct”
• “Yes, there are disproportions in how women are incarcerated. I am certain there are ways the penal system can be changed and made more human”
• “yes—and more art & education with women in prison/jail”
• “It's ridiculous & wasteful”
• “Amazing way to tell their stories. Makes me want to take some action. Thank you.”
• “knew a lot going in, but put more emotion behind it”
• “would like to learn how to be involved”
• “if you include personal stories, students can connect more”
• “I don't know the alternatives—causes for prison?”
• “Men are the highest % incarcerated. Don't we need to incarcerate less people in general?”
• “so many women in prison are mothers—people need them—and so many of those women need their mother's love”
• “it raised the question, why so many in our state?”
• “so much hopelessness—”
• “Thank you!!”
• “Yes, the injustice of the system—the women should have and be with their kids. Thanks you! Keep doing exhibits like this.”
• “Very powerful exhibit”
• “Need more ad.”
• “Thank you”
• “Mandatory rehab should be tried”
• “You are remembered”
• “Does it really help?”
• “They can be saved”
• “ways to help?”
• “very powerful”
• “need to know more about how they got there”
• “Yes! How to make this happen?”
• “this exhibit needs to go into the schools, communities and the pieces need to be a bridge for community correspondence—one could adopt a container and the message and begin the unfolding—the containers are like tissues, lace curtains, veils—stunning. Suggestion back-drop could be a solid color to show the words and vessels—containers with greater contrast. Auction off pieces for defense funds?”
• “some type of education needed + teaching for empowering some education on how to change their future for positive choices for their family, themselves to avoid every being in prison again. Classes what they would like to learn more about.”
From the Public's Survey at Open Studio Gallery during Art-a-Whirl (*N* = 140)
Question #1: “*This exhibit has increased my awareness of mass incarceration of women*”
• “I had no idea!”
• “I am a police & prison abolitionist but the depiction is powerful”
• “Moving! The scale is captivating”
• “Cannot turn away”
• “I had no idea the extent of increased incarceration in women alone”
• “No words the heart knows bless”
• “Part of this is due to a larger trend of Mass Incarceration of People of Color in the US in general, but women have specific needs that are unique to that population and often gets buried under the larger dialogue of race relations.”
• “I had no idea”
• “breathtaking!”
• “Dateline is great”
• “I was unaware”
• “Thank you for raising awareness”
• “Very powerful piece. Places the statistics in a light you can't ignore”
• “900% number is huge”
• “Why is this happening”
• “very thought provoking”
• “RAW”
• “Creates a space to challenge one's narrative”
• “I volunteer in prisons in Wisconsin”
• “Love the phrases/words”
• “Very powerful and moving”
• “I had no idea the incarceration of women was going up”
Question #2: “*This exhibit has helped me see women who are incarcerated*”
• “I need to more carefully read their write up to know more”
• “Sometimes individual stories are all we have”
• “Handwriting tracing to amplify unheard voices”
• “The statements make the women more real”
• “As a whole, there is a pattern of various uncertain emotion”
• “Clean delivery of a very deep + sad message”
• “The participation of the women in the project is a great way of having their voices heard in a way that the casual viewer will pay attention too”
• “Insightful—they have (of course) feelings”
• “Could have read more”
• “Love the tracing. It's personal”
• “I relate to the depressed feelings”
• “hard to read”
• “the small space invited me to look, making it more personal”
• “HEAR”
• “very powerful”
• “Quite sobering, gut wrenching”
• “I know more men, but one ‘felon’ made me aware we are all one
Question #3: “*This exhibit has made me realize that action is needed to reduce incarceration of wom*en”
• “[drawn heart]”
• “I had no idea the trend was increasing”
• “300%!? Cycles of violence take down prison industrial”
• “I really like that their words were handstitched. It must have taken a long time and that makes me think about how they pass time while incarcerated”
• “Will I forget? I hope not.”
• “There are better ways to deal with people who break the law. Often incarceration is a self-perpetuating cycle and it has a ripple effect into other areas of society that our government and communities are not prepared to deal with”
• “definitely”
• “Beautiful piece! Strong message! Thank you”
• “Terrible concept that we live with”
• “Judicial reform is important”
• “politics must change laws!”
• “U.S. prison system is appalling. New form of slavery.”
• “Are they wrongly incarcerated?”
• “How?”
• “Brilliantly done, compassionate”
• “INCARCERATION DOES NOTHING BUT SLOW SOCIETY'S PROGRESSION”
• “5% world population, 25% of incarcerated. The land of the free”
• “I'm also concerned that sexism keeps women from being viewed as a threat and wonder if the increased incarceration is part of leveling ‘power’ balance. Though I'd rather see reduced incarceration for everyone”
• “Lovely!”
• “Way too easy to ignore the realities. Your exhibit stirs a desire for action. Keep their reality alive.”
• “THANK YOU”
• “A glorious, significant mission”
• “A lovely way to depict something so heartbreaking. Thank you.”
• “I would love to help, but how?”
From the Public's Survey at Open Studio Gallery during Open Casket (*N* = 64)
Question #1: “*This exhibit has increased my awareness of mass incarceration of women*”
• “I had no idea about the #s”
• “Very sad”
• “I thought the embroidered names were very thought provoking”
• “creatively stretched my view of the horror of being incarcerated”
• “it helps feel it”
• “Excellent topic”
• “No idea it has increased dramatically”
• “Thanks!”
• “900% → what!?”
• “Especially about women in MN—I had no idea”
• “the artist's statement really helped contextualize the pieces”
• “I did not know the extent to which female incarceration rates had grown.”
• “I'd like more info on what crimes these women are accused of + of their demographics.”
• “The rate increase and how US compares to rest of the world”
• “WOW”
Question #2: “*This exhibit has helped me see women who are incarcerated*”
• “I'd like to know the demographics of their accusers as well.”
• “w/the third eye”
• “yes their language is really manifested in form”
• “their voice came alive in a way that can't be done elsewhere”
• “short fragments of statements are powerful esp. with the tendency towards short attention span of audiences”
• “Painful to look @”
• “I've know several women who have been incarcerated. This is poignant.”
• “Yes, & now I'm more curious”
• “Written explanation influenced me”
Question #3: “*This exhibit has made me realize that action is needed to reduce incarceration of wom*en”
• “what a statement!”
• “Our system sucks. Grateful for your work—very powerful—thank you”
• “these women need to be humanized”
• “incarceration is an issue for all, however the massive increase of women is alarming”
• “action is needed for all genders”
• “Love this work”
• “Please keep going!”
• “tragic, broken system—the next generation suffers”
• “Very inspiring”
• “You lead with your work”
• “Yes, absolutely, why not State & Federal prison”
• “Thank you for taking the time/energy to make important work!”
• “Thank you for what you do! (& express!)”
• “And that men & women should be re-integrated by more individualized treatment!”
• “I'm appalled about these statistics and I think the penal system needs to conform to human decency rather than disposal.”
• “the use of shadow is very effective”
• “Thank you for your craft”
• “Very touching”
• “The messages are powerful. Attention grabbing presentation.”
• “Thank you for the awareness”
From the Public's Survey at Walker Public Library (*N* = 62)
Question #1: “*This exhibit has increased my awareness of mass incarceration of women*”
• “Very well done and displayed by the artist”
• “I can't see any correlation between the art/incarceration”
• “I had no idea!”
• “Beautiful but also so sad”
• “Very eye catching”
• “God's help is needed” *[$1.00 Dollar bill enclosed]*
• “I had no idea the rate of increase was so high!”
• “The display shrinks the view”
• “Only because of this question, + then rereading the ‘stop look art’ sheet”
• “It's blatant racism”
• “This work needs national attention!”
• “I had no idea it was this high”
• “Given the numbers/stats”
• “The facts on the display were helpful + translations +/or audio would be good too”
Question #2: “*This exhibit has helped me see women who are incarcerated*”
• “this is beautiful!”
• “Not seeing any tie between art + crim. justice system”
• “They become real”
• “The writing is touching and effective”
• “Beautiful humanity”
• “The handwriting is difficult to read so I learned via artists instead of incarcerated women”
• “the white tissue does not add humanity & covers writings”
• “Agree somewhat—the mssgs that I can see are a window to their humanity”
• “An amazing exhibit”
• “Very beautiful concept!”
• “This work needs national attention!”
• “I find it very good also how one cannot see all the words. Almost as if the voices get lost in our criminal justice system. And they spill over the case, I find that really superb”
• “Not literally, but definitely figuratively”
• “How can we read/see more?”
• “The hand embroidery feels very personal”
Question #3: “*This exhibit has made me realize that action is needed to reduce incarceration of wom*en”
• “Would need more info re: the crimes to make a comment. Also, would be good to know what % is minority & what % Caucasian”
• “Absolutely stunning”
• “Again no example of incarceration tied to art”
• “great”
• “Important work!”
• “what are the alternatives”
• “yes”
• “All need help sometime”
• “No call to action was explicitly stated. Where do I go to help? What do I tell my representatives?”
• “Not enough stated research on WHY”
• “Because of the facts section in the artist's stmt and the above. I needed something besides this questionnaire to catch my eye + tell me what the exhibit's about. I am sensitive to this topic + still don't catch it w/out this questionnaire”
• “Good Job, Well Done!”
• “and of black men as well. This is powerful. Thank you for showing it.”
• “No person should be incarcerated.” ^***^ (3 x strongly disagree)
• “It has made me aware + interested in see the reasons + other solution to incarceration”
• “the exhibition ‘starts' the thinking on this subject: good!”
• “Like to talk more”
• “Some going from one life-trap to another; others getting relief or other kinds of help. Thank you for doing this!”
• “Thank you, for this very meaningful work”
• “The artist's care & concern for each inmate shines through”
• “Powerful layering of pieces! Illustrates the horrific dehumanizing element of incarceration.”
• “Doesn't offer ways to be active”
• “This work needs national attention!”
• “Good concept”
• “No action steps offered—I already recognize the issue”
• “Lovely”
• “Mass incarceration is a serious issue!”
From the Public's Survey at frame.de.galerie, Amersfoort, NL (*N* = 15) 9/22/2019
Question #1: “*This exhibit has increased my awareness of mass incarceration of women in the US*”
• “I was already interested in this topic”
• “Shocking number”
• “Was a real eye opener”
• “I'm feeling it in my bones now”
• “I found especially the amount that made it beautiful also impressive”
Question #2: “*This exhibit has helped me see women who are incarcerated*”
• “Through their own words it is taken in”
• “I would love to help, but how?”
• “You will forever be in my heart”
• “So much hopelessness”
• “Extremely painful”
Question #3: “*This exhibit has made me realize that action is needed to reduce incarceration of wom*en”
• “I will know that with more information on the reasons for incarceration”
• “Where to start?”
• “There is a need to change the mind of people how their imprisonment can be good. Freedom heals, bars don't”
• “Short film = excellent documentation. Photos are not sharp enough”
• “Though women! Very impressive (I didn't know it was this bad). Such a positive action of the artist. Much success!”
• “Impressive and so beautiful”
• “Insufficient legal aid”
• “Superb exhibit!”
• “Absolutely. There is still much to do!”
• “It is unbelievable what society does to these women even if they have done something wrong”
• “There is a need to change the mind of people who think imprisonment can be good. Freedom heals, bars don't”
• “We have listened to you”
From the Public's Survey at the Phipps Galleries, Hudson, WI (*N* = 57)
Question #1: “*This exhibit has increased my awareness of mass incarceration of women*”
• “I have worked with women prisoners—while this exhibit is moving, I'm sadly already aware of mass incarceration”
• “I had no idea”
• “Stats were eye opening”
• “There has to be a better way than incarceration”
• “Beautiful”
• “Don't know how many women in prison”
• “people”
• “You can't ever tell that's the subject matter”
Question #2: “*This exhibit has helped me see women who are incarcerated*”
• “Reading snippets of their words was powerful”
• “Creative”
• “What a beautiful gift you've given to these women and to us.”
• “You picked volume over images to create strongly impactful pieces”
• “yes & feel”
Question #3: “*This exhibit has made me realize that action is needed to reduce incarceration of wom*en”
• “See above. What interesting work. I can imagine how meaningful your visits are to the women!!”
• “powerful—thank you for letting these voices be heard”
• “Outstanding ‘Configuration’—bound in chains!?”
• “Thank you!”
• “Thank you! From a social worker”
• “Aware. Supportive”
• “60% not convicted—don't get that? Flee risk? Where are the children?”
• “Yes! They are mothers!”
• “YES DO IT!”
• “Talented”
• “Thank you. Important work. We need to support women more”
• “My partner was an incarcerated woman. Thank you for giving her a voice. The stitching piece is extremely powerful.”
• “I already understood this.”
• “Thanks!”
• “Thank you”
• “What factors drive these numbers?”
• “KNOW WE NEED TO REDUCE INCARCERATION FOR MANY WOMEN MEN, IMMIGRANTS…”
• “people”
• “Don't break the law”
• “I knew that anyways. Stop with subtle implications.”
• “You give no facts to support your hypotheses”

Voluntary comments of the public show a deeper understanding on viewing the artwork. There is a universal pathos expressed in the sentiments that inspires not only empathy for the women's sad plight but also a sense of the shared, personal feelings of regret, longing, separation, alienation, helplessness, and even entrapment that we all experience to some degree as human beings in this unjust world.

## Discussion

The principal finding of this analysis was that visual art involving original handwritings provides benefits both to women in the jails participating in the project and to individuals in society who were able to view the resulting art installations. The artist who was granted access to work with women residents at the jails invited women residents to explore their own handwritings and optionally share with a greater audience what is important to them. It may have provided a form of validation of their situation, and as demonstrated by the return of the surveys, was viewed favorably. There is ample evidence that creative forms of art among individuals in care facilities serve an important purpose for facilitating shared social connectedness among residents with an improved sense of well-being.^1^ Among individuals with criminal justice involvement, use of participation in drama projects has shown favorable effects in altering the emotional state related to traumatic experiences and improves capacities to regulate interpersonal relations.^9^ Similar favorable effects among individuals experiencing incarceration have been shown with meditation, as a means of fostering mindfulness-based stress reduction.^10^ The women who participated in the creation of handwritings as visual art often commented in their work on their roles in motherhood, and the stresses that were created by their situation for dependents. Considering that 80% of women in jail are mothers and most are primary caretakers of the children, it is a critical problem to address how incarcerated mothers' parenting is intertwined with their health and sense of well-being.^5^

The notion that art can be a catalyst for positive change, among both women locked up in jails and the public, is timely. Incarceration rates among women in Minnesota have increased logarithmically over the past three decades, and considering the recent events involving COVID-19, marginalized members of our community in jails represent the most vulnerable individuals.^4^ The original handwritings of the women experiencing incarceration were traced through hand-stitching by the artist and embedded into individual sculptures. Site-specific installations were created at various public venues, and the response by viewers participating in the surveys was favorable. Many of the handwritings evoke emotion, inciting awe and wonder, which is an important tool to elicit empathy.^8^ This highlights the importance of shifting the paradigm of incarceration from “tough on crime” rhetoric, to more inclusive messages, particularly related to the inherent humanity of alleged offenders.^11^ It is very likely that the production of art validates many emotions of marginalized individuals who have suffered from the injustices of our legal system as well as the inequities created by poverty within communities.^6^ Arts-informed approaches may be one of the most valuable means of engaging the public in disparities of marginalized members of the community.^12^

Creating change to address mass incarceration requires many elements. Incarceration of women in Minnesota has increased ninefold over the past three decades, with a negative ripple effect through affected communities. The divide between women experiencing incarceration and society has led to a polarization, which can be addressed through the arts and its related expression of human empathy. These systems of punishment, control, and isolation should be abolished and replaced with human investments that allow all to thrive while being held accountable. It is through the arts that empathy can be the instrument of change.

In summary, we have shown that art, both in its creation of handwritings by women experiencing incarceration and in its amplified showings at public installations, offers great benefits. The funding sources through philanthropy are limited and unfortunately, many artists are unable to sustain their role in continuing their craft within marginalized communities. We hope that public policies will support marginalized individuals who suffer from inequities in the legal system with tools of creative self-empowerment that actively and holistically embrace their human spirit so that in time, in tandem with other efforts in the areas of social welfare, jobs, health, food, education, housing, and environment, inequities become obsolete with a just future for all. Art can be a great tool toward one's self-fulfillment and purpose under adverse circumstances.

## Abbreviation Used

BIPOC: Black, Indigenous, People of Color

## References

[1] Windle G, Caulfield M, Woods B, Joling K How can the arts influence the attitudes of dementia caregivers? A mixed-methods longitudinal investigation. Gerontologist 2020;60:1103–1114 32447369 10.1093/geront/gnaa005 PMC7427486

[2] Feen-Calligan H, Moreno J, Buzzard E Art therapy, community building, activism, and outcomes. Front Psychol 2018;9:1548 30333764 10.3389/fpsyg.2018.01548 PMC6176654

[3] Di Lellio A, Rushiti F, Tahiraj K “Thinking of You” in Kosovo: Art activism against the stigma of sexual violence. Violence Against Women 2019;25:1543–1557 31506019 10.1177/1077801219869553

[4] Reinhart E, Chen D. Incarceration and its disseminations: COVID-19 pandemic lessons from Chicago's cook county jail. Health Aff (Millwood) 2020:101377hlthaff202000652 10.1377/hlthaff.2020.00652 32496864

[5] Kennedy SC, Mennicke AM, Allen C ‘I took care of my kids': Mothering while incarcerated. Health Justice 2020;8:12 32504229 10.1186/s40352-020-00109-3 PMC7275483

[6] Gottlieb A. Incarceration and relative poverty in cross-national perspective: The moderating roles of female employment and the welfare state. Soc Serv Rev 2017;91:293–318 29104322 10.1086/692357 PMC5663300

[7] Chen JT, LaLopa J, Dang DK Impact of Patient Empathy Modeling on pharmacy students caring for the underserved. Am J Pharm Educ 2008;72:40 18483606 10.5688/aj720240 PMC2384215

[8] Paulson S, Sideris L, Stellar J, Valdesolo P Beyond oneself: The ethics and psychology of awe. Eval Program Plann 2013;36:165–171 32519393 10.1111/nyas.14323

[9] Mundt AP, Marín P, Gabrysch C, Sepúlveda C, Roumeau J, Heritage P Initiating change of people with criminal justice involvement through participation in a drama project: An exploratory study. Front Psychiatry 2019;10:716 31632309 10.3389/fpsyt.2019.00716 PMC6785769

[10] Himelstein S. Meditation research: The state of the art in correctional settings. Int J Offender Ther Comp Criminol 2011;55:646–661 20332328 10.1177/0306624X10364485

[11] Gottlieb A. The effect of message frames on public attitudes toward criminal justice reform for nonviolent offenses. Crime Delinq 2017;63:636–656 28943646 10.1177/0011128716687758 PMC5606148

[12] Parsons J, Heus L, Moravac C Seeing voices of health disparity: Evaluating arts projects as influence processes. Eval Program Plann 2013;36:165–171 22469339 10.1016/j.evalprogplan.2012.03.003

